# Nursing workload, staffing qualifications, and nursing care costs associated with hospital-acquired pressure ulcer days in the ICU: a retrospective cohort study

**DOI:** 10.3389/fpubh.2026.1836283

**Published:** 2026-09-09

**Authors:** Beata Wieczorek-Wójcik, Anna Milewska, Rafał Kobielak, Remigiusz Kozłowski, Petre Iltchev, Dorota Kilańska

**Affiliations:** 1 Division of Nursing and Medical Rescue, Pomeranian University in Słupsk, Słupsk, Poland; 2 Division of Nursing Management, Institute of Nursing and Midwifery, Medical University of Gdańsk, Gdańsk, Poland; 3 Department of Biostatistics and Medical Informatics, Medical University of Białystok, Białystok, Poland; 4 Ceynowa Hospital in Wejherowo, Pomeranian Hospitals LLC in Gdynia, Gdynia, Poland; 5 Department of Coordinated Care, Medical University of Lodz, Lodz, Poland; 6 Department of Management and Logistics in Healthcare, Medical University of Łódź, Łódź, Poland; 7 Division of Coordinated Care, Institute of Nursing and Midwifery, Medical University of Gdańsk, Gdańsk, Poland

**Keywords:** health care costs, missed nursing care, patient safety, pressure ulcer, risk management, staffing levels, workload indicators

## Abstract

**Background:**

Hospital-acquired pressure ulcers (HAPUs) are frequent and costly adverse events. Inadequate staffing and missed nursing care may contribute to preventable complications and higher healthcare costs.

**Objective:**

The aim of this study was to assess the associations between nursing workload, staffing qualifications, and nursing care costs and the occurrence of hospital-acquired pressure ulcer days in ICU patients using retrospective cohort data.

**Methods:**

This study included 1,461 ICU care days from 2019 to 2022. Variables examined were Nursing Hours per Patient Day (NHPPD), patient-days, nursing educational structure, and care complexity assessed using TISS-28. Logistic with Newey–West standard errors evaluated associations of NHPPD and patient-days with HAPU days. Nursing care costs were analyzed separately using non-parametric tests comparisons.

**Results:**

A total of 81 days with HAPU were identified (5.4% of). Each one-hour increase in NHPPD was associated with 9% lower odds of a HAPU Day (OR = 0.91; 95% CI: 0.83–1.00; *p* = 0.035), whereas each additional patient-day was associated with 19% higher odd (OR = 1.19; 95% CI: 1.01–1.40; *p* = 0.047). Nursing care costs varied significantly across study years. The highest values observed in year 2022 (*p* < 0.001). Educational structure of the nursing staff, nursing care costs and complexity of care was not statistically significantly associated with HAPU occurrence in this study.

**Conclusion:**

Nursing workload indicators, particularly NHPPD and patient demand, were significantly associated with HAPU occurrence in the ICU. Adequate nursing staffing may contribute to reducing preventable adverse events and improving patient safety in intensive care settings.

## 1 Introduction

Findings from studies conducted in intensive care remain inconclusive. In environments characterized by high team interdependence, rapid interventions, and highly dynamic clinical situations, practical competencies may play a more significant role than formal educational level (1, 2). In addition, substantial clinical variability and workload pressure related to patient-days may mask the effect of qualification structure on patient outcomes (3).

Despite the growing number of publications, most studies rely on self-reported assessments by nurses, which limits the ability to capture the true dynamics of workload. Only a limited number of studies utilize objective hospital system data, NHPPD records, or real-time information on patient numbers and clinical complexity (TISS-28) (4–7).

Hospital-acquired pressure ulcers (HAPU) are among the most common (8) and at the same time the costliest adverse events (9), the occurrence of which strongly depends on the availability and quality of nursing care. Since the time of Florence Nightingale, who pointed out that patient injuries result not from the disease itself but from inadequate nursing care (10), pressure ulcer prevention has remained one of the fundamental areas of nursing responsibility. Today, HAPUs are classified as one of the key nursing-sensitive indicators, reflecting the quality of care and the effectiveness of the healthcare system (11–14).

The incidence of HAPUs in intensive care units (ICUs) ranges from 10 to 25%, which is attributable to the severity of patients’ conditions, impaired perfusion, immobilization, and the therapies applied, including vasopressor and renal replacement therapies (7, 15, 16).

International analyses have demonstrated that higher NHPPD levels are associated with lower mortality, shorter lengths of hospital stay, and a reduction in adverse events (17, 18). Studies by Aiken et al. (19) showed that each additional patient per nurse increases the risk of adverse events by 7%, while Needleman et al. (20) demonstrated that staffing shortages during shifts are associated with an increased risk of complications. However, an increase in the number of patients assigned to a nurse leads to greater workload, higher stress levels, and a higher frequency of missed care events (9, 21, 22). An increasing body of evidence suggests that nursing workload may influence patient outcomes through mechanisms such as missed nursing care, defined as elements of required care that are omitted, delayed, or incomplete because of insufficient nursing resources or excessive workload (23–25). Missed care includes, among others, delays in patient repositioning, inadequate hygiene, lack of skin assessment, or failure to implement recommended preventive interventions. It is recognized as an early warning indicator of declining quality of care (26). Although missed care has been proposed as an important mediator between staffing and adverse patient outcomes, including HAPUs (13, 27, 28), it was not directly measured in the present study. Instead, this study focuses on organizational factors that have consistently been identified as upstream determinants of missed care, including nursing workload, staffing levels, and qualification structure.

At the same time, an important factor modifying the quality of care may be the competency structure of the nursing team. Higher levels of education and nursing specialization are associated with a greater ability to apply evidence-based practice, improved risk recognition, and the timely implementation of preventive interventions (29–31). Studies have shown that a higher proportion of nurses with advanced education is associated with lower mortality, shorter ICU length of stay, and more effective prevention of HAPUs (32–35).

Another important limitation of previous organizational studies is the use of a patient or a hospitalization episode as the unit of analysis. Analyzing the ICU care day as the observational unit allows organizational exposures and HAPU occurrence to be assessed within the same temporal framework. Although HAPU develops in individual patients, the organizational conditions influence its occurrence—such as staffing, workload, patient occupancy, and care demand—vary daily.

There is also a lack of studies analyzing the ICU Day as the statistical unit, although this approach is methodologically appropriate for evaluating organizational determinants of patient outcomes. The ICU Day was selected as the unit of analysis because the primary objective of the study was to investigate organizational rather than patient-level factors associated with HAPU occurrence. Organizational conditions such as nursing workload, staffing availability, and patient occupancy, care complexity, workforce qualification structure, and nursing care costs are routinely recorded and may vary daily (36–38). Accordingly, the ICU care day provides a common temporal framework for evaluating these organizational exposures and HAPU occurrence. In the present study, a HAPU Day was defined as a 24-h ICU care day during which at least one hospital-acquired pressure ulcer was diagnosed in any patient hospitalized in the ICU, regardless of pressure injury stage. Because consecutive ICU care days may share patients and organizational characteristics, temporal dependence between observations was accounted for in the statistical analysis using Newey–West robust standard errors.

In Poland, there is a lack of studies assessing the impact of nursing workload, demand for care, and the qualification structure of nursing staff on the risk of HAPU in intensive care units. To date, there is limited evidence from studies using routinely collected hospital information system (HIS) data to examine associations between NHPPD, patient-days, nursing workforce characteristics, and HAPU occurrence at the ICU-day level.

In the present study, HAPU Day was defined as a 24-h period during which at least one hospital-acquired pressure ulcer was diagnosed in any patient hospitalized in the ICU, regardless of pressure injury stage. This approach enabled workload, staffing, care demand, and HAPU occurrence to be analyzed at the same temporal level. Consequently, this approach is better suited to evaluating organizational factors associated with HAPU occurrence (39).

Given the multi-factorial etiology of HAPU, this study adopted a multi-variable approach, enabling the simultaneous assessment of the effects of workload, staff qualification structure, and care demand resulting from care complexity (TISS-28) on the risk of a HAPU days (40–49). Several decades of research have demonstrated the economic and clinical value of nursing care in improving healthcare quality and preventing adverse events. Inadequate staffing and missed nursing care have been associated with increased rates of preventable complications, which account for a substantial proportion of hospital expenditure. Previous estimates suggest that approximately 15% of hospital costs are related to the treatment of adverse events and their consequences. In the Polish regulatory context, nurse staffing in intensive care is determined on the basis of patient care needs, nursing workload, care complexity, and staff qualifications (50). These variables were therefore selected not only because of their relevance in the international literature, but also because they reflect the organizational criteria used in Polish ICU staffing regulations and recommendations.

The present study did not directly assess individual preventive nursing interventions, missed nursing care, or patient-level clinical risk factors. Instead, it examined objective organizational conditions—including NHPPD, patient-days, nursing staff qualification structure, care complexity, and nursing care costs—that may influence the environment in which preventive care is delivered.

The conceptual framework of the study is presented in Supplementary Figure 1. It illustrates the organizational factors examined in relation to HAPU Day occurrence at the ICU care-day level, together with calendar-year adjustment and potential residual confounding by unmeasured patient-level clinical factors. The framework represents the associations examined in the study and does not imply causal relationships.

The aim of this study was to assess the associations between nursing workload, staffing qualifications, and nursing care costs and the occurrence of hospital-acquired pressure ulcer days in ICU patients using retrospective cohort data.

## 2 Methods

### 2.1 Study design

This retrospective cohort study was designed in accordance with the STROBE methodology. The observed population consisted of 1,461 ICU care days provided to patients in the Intensive Care Unit of the Specialist Hospital in Wejherowo. The analysis simultaneously included multiple exposure factors which, based on literature and clinical, organizational assumptions, may influence the risk of a day with HAPU. The unit of analysis was the ICU care day, defined as a 24-h organizational observation period. The daily patient census was recorded once each day at 07:00. Patients who remained in the ICU at the time of the morning census were included in the patient-day count for that day. Patients admitted and discharged between two consecutive census measurements were not represented in that specific daily census.

The observed event was HAPU Day. A HAPU Day was recorded when at least one hospital-acquired pressure ulcer was documented in any patient hospitalized in the ICU during that day; otherwise, the day was classified as a non-HAPU Day.

**Figure 1:**
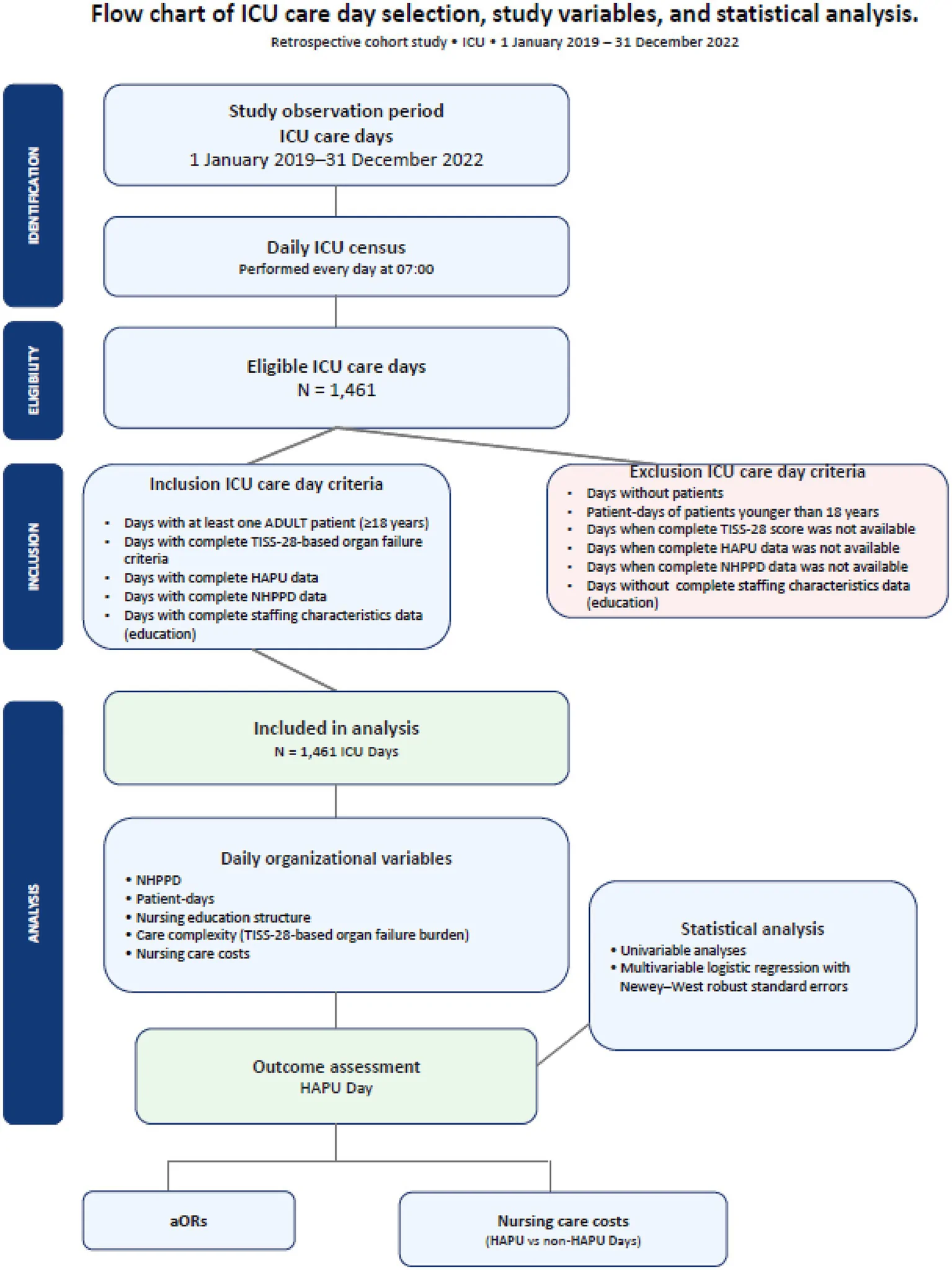
Flow chart of ICU care day selection, study variables, and statistical analysis.

All organizational variables, including nursing workload (NHPPD), patient-days, nursing care costs, educational structure of the nursing workforce, care complexity, and organ failure categories, were aggregated and measured at the daily ICU level (Figure 1).

Data were obtained from three routinely collected hospital information sources: the electronic health record (HIS), the nursing staffing database, and the hospital payroll and human resources system.

### 2.2 Setting

The study took place between 1.01.2019 and 31.12.2022 in the Intensive Care Unit (10 beds) of the Specialist Hospital in Wejherowo (study hospital) in the Pomeranian province. The hospital provides intensive care services for about 330,000 residents of 2 counties. This represents 1/7th of the population of the Pomeranian province and can be considered a reference population. The hospital provides specialized care at the third reference level of the hospital network for 20% of the region’s population, offering state-of-the-art services in specialized medical facilities. The unit is a medical-surgical intensive care unit, providing treatment to patients in life-threatening conditions caused by potentially reversible failure of one or more major organ systems. It admits patients with respiratory failure requiring mechanical ventilation, as well as patients requiring renal replacement therapy. Approximately 300 patients are hospitalized annually, including cardiac surgery, general surgery, and neurosurgical patients. The unit employs 51 nurses, including 29 nurses with specialist qualifications and 27 nurses holding a master’s degree in nursing.

### 2.3 Population

This retrospective cohort observational study analyzed 1,461 consecutive ICU care days recorded between 2019 and 2022 in the Intensive Care Unit of the Specialist Hospital in Wejherowo from the Hospital Information System (HIS), nursing staffing records/schedules, and hospital payroll and human resources records. No participants were prospectively recruited or contacted, and no additional patient- or nurse-reported data were collected for the purposes of the study.

Each ICU care day represented a 24-h organizational observation period. The ICU Day was selected as the analytical unit because all organizational exposure variables (NHPPD, patient-days, staffing characteristics, nursing costs, and care complexity) were routinely measured and managed at the daily unit level. All eligible ICU care days were included in the analysis, and no ICU care days were excluded.

Inclusion criteria ICU care day: The unit of analysis was the ICU care day, defined as a 24-h organizational observation period. All 1,461 consecutive ICU care days with complete routinely collected data on patient census, complete NHPPD data, staffing characteristics (education), care complexity (days with complete TISS-28-based organ failure criteria), and HAPU occurrence were included. No eligible ICU care days were excluded from the analysis. Pressure ulcers present on ICU admission were considered pre-existing and were not classified as HAPU outcomes. The source population comprised all ICU days with adult patients (≥18 years) hospitalized in the ICU between 2019 and 2022.

Exclusion criteria ICU care day: ICU care days excluded from the analysis were days without patients. No eligible ICU care days were excluded from the analysis. ICU care days with patients younger than 18 years were outside the defined source population; days when complete TISS-28 score and NHPPD data were not available; Days without complete staffing characteristics data (education). Pressure ulcers present on ICU admission were not classified as hospital-acquired pressure ulcers and were therefore not counted as outcomes.

### 2.4 Variables

The data formed a continuous time series, and consecutive ICU care days were dependent. The average ICU length of stay was 21 days, meaning that the same patients could contribute information to multiple consecutive ICU care days. During this period, patients’ clinical status and care complexity could change. For example, a patient initially classified as being at risk of organ failure could subsequently develop organ failure, resulting in changes in the distribution of care complexity across consecutive ICU care days. The structure of the study data is presented in Table 1.

**Table 1:** The structure of the study data.

ID Day	Date	Patient-days	No. nurses with level of education	NHPPD	No. patients with organ failure with levels of category			No. of HAPU	HAPU Day
All ICU care days (4 years) 2019-2022 4*365 + 1 = 1,461		*n*	*n*	*n*	Two or more organ failures	Single organ failure	Risk of organ failure	*n*	0 – No 1 - Yes
1	1.01.2019	7	Master in Nursing and specialization, Bachelor in Nursing and specialization, Master in Nursing, Bachelor in Nursing, Specialization, Other Registered Nurses.	17.14	0	4	3	0	0
2	2.01.2019	7		17.14	0	6	1	0	0
…	…	…		…				…	…
114	24.04.2019	8		15.00	4	3	1	1	1
…	…	…		…				…	…
1,461	31.12.2022	6		16.00	4	2	0	0	0

The following categories were distinguished among the variables: observed event, exposure variables, confounding factor, exploratory variable.

#### 2.4.1 The observed event: HAPU day

To assess HAPUs and their severity, the EPUAP classification was used. A pressure ulcer was defined as a localized injury to the skin and/or underlying tissue, usually occurring over a bony prominence as a result of pressure or a combination of pressure and shear forces (51). Data on HAPUs were collected from the electronic patient records (HIS – CLININET). In this study, a HAPU Day was defined as a day of care on which at least one hospital-acquired pressure ulcer was diagnosed, regardless of its stage classification.

#### 2.4.2 Organizational exposure: nursing workload – NHPPD and patient-day

In Poland, ICU nurse staffing is regulated by the Ministry of Health (52). The minimum staffing level is based on a fixed nurse-to-bed ratio (2.2 full-time equivalent nursing positions per ICU bed). For the purposes of these regulations, an anesthesia and intensive care nurse is defined as a registered nurse who has completed, or is undertaking, a specialization in anesthesiology and intensive care nursing, or who has completed a qualification course in this field. Since 2016, the organizational standard has additionally required that staffing decisions consider patient care complexity, including the number of organ failures. Therefore, the present study included organizational indicators routinely used in ICU staffing management.

Workload was monitored using the patient-day indicator. A patient-day was defined as the number of patients present in the ICU during a 24-h organizational observation period. The daily patient census was recorded once each day at 07:00, and this census formed the basis for calculating the patient-day indicator.

NHPPD reflects the amount of nursing care available per patient-day based on the actual number of nursing hours provided by all categories of registered nurses (53–55). NHPPD was used additionally as an indicator of the nursing resources available in relation to daily patient demand. In the present study, NHPPD was calculated using the actual number of nursing hours per patient-day provided during each 24-h period. Daily staffing was organized in accordance with the Polish standard of 2.2 full-time equivalent anesthesia and intensive care nurses per intensive care bed.

#### 2.4.3 Organizational exposure: educational structure of the nursing workforce

During the study period (2019–2022), the ICU nursing workforce comprised registered nurses with different educational qualifications and postgraduate training. To the present study, nurses were classified into six educational and professional categories:

1. Registered nurses (RNs) with secondary education (vocational studies).
2. RNs with secondary education and a specialization in anesthesiology and intensive care nursing.
3. RNs with a bachelor’s degree (BSN).
4. RNs with a master’s degree (MSN).
5. RNs with a bachelor’s degree (BSN) and a specialization in anesthesiology and intensive care nursing.
6. RNs with a master’s degree (MSN) and a specialization in anesthesiology and intensive care nursing.

Nursing hours provided by all six groups were summed to calculate daily NHPPD. Annual NHPPD values and the overall mean NHPPD for the four-year study period were subsequently calculated. In addition, the proportion of nurses in each educational category was calculated for each ICU Day and analyzed as an organizational exposure variable.

#### 2.4.4 Clinical exposure: care complexity

Demand for care was calculated based on care complexity for all hospitalized patients with a risk of or diagnosed organ failure, using three categories of care complexity (intensity) according to the TISS-28 scale, which classifies ICU patients into four categories (56). The study focused on variables related to risk and the number of organ failures in individual patients on a given day, due to data collection standards required by ICU regulations.

Care complexity (care intensity) was daily assessed using the TISS-28 classification and the corresponding organ failure criteria routinely applied in Polish ICUs (52, 57).

Six organ systems were evaluated: respiratory, cardiovascular, renal, nervous, gastrointestinal, and musculoskeletal. For each organ system, predefined TISS-28 criteria and threshold scores were used to determine the presence of organ failure. Patients were subsequently classified into three categories according to organ failure burden: at risk of organ failure, one organ failure, or two or more organ failures. All clinical data were recorded in the hospital information system (HIS–Clininet) and verified against daily nursing documentation. Detailed classification criteria and threshold scores for each organ system are presented in Supplementary Tables 1, 2 (56).

#### 2.4.5 Exploratory variable: nursing care costs

The annual cost of nurses’ wages was obtained from the hospital’s HR and payroll system. The dataset contained monthly salary data for nurses employed in 2019–2022. Salary costs included total employment-related expenditures, including base salary, overtime payments, and employer-related labor costs.

The hourly cost of nursing care was calculated separately for each month by dividing total monthly nursing salary expenditure by the total number of nursing care hours recorded in the same month. The resulting monthly average hourly labor cost was then applied to each ICU Day within that month. Daily nursing care cost was calculated by multiplying the monthly average hourly labor cost by the actual number of nursing care hours worked on the corresponding day. Thus, day-to-day variation in nursing care costs reflected variation in the number of nursing hours provided, while the hourly labor cost remained constant within each month.

Nursing salary costs were calculated in Polish zloty (PLN) and converted into euros (EUR) using the average annual exchange rates published by the National Bank of Poland for 2019–2022 (4.30, 4.44, 4.58, and 4.69 PLN/EUR, respectively). For the pooled comparison of mean costs between HAPU and non-HAPU Days across the entire four-year period, the average exchange rate for 2019–2022 (4.50 PLN/EUR) was applied.

#### 2.4.6 Counfounding factor - years

The study covered a four-year observation period. Calendar year was included in the analysis as either a stratification variable or an adjustment variable.

### 2.5 Data sources

Data on ICU Days during the four-year observation period (2019–2022) were obtained from three routinely collected hospital data sources:

The Hospital Information System (HIS – CLININET), which records patient data including demographic characteristics, admissions and discharges, ICD-10 diagnoses (Appendix 1), TISS-28 scores indicating the risk of or presence of organ failure, pressure ulcer risk assessment, and the occurrence of HAPUs. Supplementary Table 3: characteristics of patients hospitalized in the period 2019–2022 (*N* = 1,056).

An Excel-based nursing staffing monitoring system (author-developed), which records daily information on direct and indirect nursing care activities, Nursing Hours per Patient Day (NHPPD), daily patient census (patient-days), the number of patients at risk of organ failure, patients with one organ failure, patients with two or more organ failures, and the occurrence of HAPU Days and non-HAPU Days.

The hospital payroll and human resources system, which provided monthly data on nurses’ salaries, including base salary, overtime payments, and employer-related labor costs. These data were used to calculate daily nursing care costs.

### 2.6 Bias

#### 2.6.1 Data source and measurement-related bias

The study is subject to potential sources of systematic bias. The analysis included data from a single intensive care unit, which may limit the generalisability of the findings to other institutions and patient populations.

The patient census was recorded once daily at 07:00. Therefore, patients admitted and discharged between two consecutive census measurements may not have been fully captured in the daily patient-day indicator. This may have introduced measurement error and potentially underestimated short-term fluctuations in patient volume and nursing workload. However, the same census procedure was applied consistently throughout the study period, reducing the risk of differential measurement across ICU care days.

In addition, the data were derived from routinely collected electronic records, including the HIS and nursing staffing registry, which entails a risk of incomplete or inaccurate entries. Variability in pressure ulcer diagnosis and documentation may have resulted in misclassification or underestimation of HAPU occurrence. Misclassification of care complexity based on the selected TISS-28 criteria cannot be excluded.

#### 2.6.2 Residual confounding

Important patient-level risk factors for pressure ulcer development, including age, comorbidities such as diabetes or vascular disease, immobility, nutritional status, APACHE II or SOFA scores, and Braden Scale scores, were not included in the analysis. This reflects the organizational focus of the study and the structure of the routinely collected data available for nursing staffing assessment in Polish ICU practice.

In the Polish ICU regulatory context, patient care demand is assessed primarily through organ failure burden as an indicator of care intensity, including TISS-28-based categories, rather than through comprehensive patient-level pressure ulcer risk models. Nevertheless, the absence of individual clinical risk factors may have resulted in residual confounding. Therefore, the observed associations between organizational exposures and HAPU Day occurrence should be interpreted cautiously.

#### 2.6.3 Nursing care cost measurement

Nursing care costs were used as an organizational indicator of nursing labor resource use and should not be interpreted as a direct measure of the quality or intensity of bedside nursing care. The study did not directly measure missed nursing care or omitted preventive interventions; therefore, the observed associations between nursing care costs and HAPU occurrence cannot be used to infer mechanisms involving missed or omitted care. Nursing care costs were aggregated across the 2019–2022 period without adjustment for inflation or temporal changes in labor costs. Consequently, year-to-year differences related to salary increases, overtime payments, and the COVID-19 pandemic may not have been fully captured. The estimates included nursing labor costs only and did not represent the broader economic burden of HAPU, such as prolonged hospitalization, treatment materials, or physician-related costs. Furthermore, daily nursing care costs were estimated using average hourly labor costs rather than patient-level resource utilization, which may have resulted in under- or overestimation of actual costs.

#### 2.6.4 Temporal dependence of ICU care-day observations

The data formed a continuous time series, and consecutive ICU care days were correlated. Depending on the structure of the dataset, appropriate analytical approaches may include generalized estimating equations, mixed-effects models, or generalized linear models with robust standard error correction. In the present study, temporal dependence was addressed using Newey–West heteroskedasticity- and autocorrelation-consistent (HAC) robust standard errors, as described in the Statistical Methods section.

### 2.7 Study size

All observation ICU care days (*N* = 1,461) from the period 2019–2022 were included in the analysis.

### 2.8 Quantitative variables

Two quantitative variables were analyzed in this study: Nursing Hours per Patient Day (NHPPD) and daily nursing care costs. Both variables were analyzed as continuous variables without categorization or transformation. Because the distributions of NHPPD and nursing care costs were skewed, non-parametric statistical methods were used for group comparisons.

### 2.9 Statistical methods

In the first step, the effect of the observed years was analyzed as a potential confounding factor. In the second step, uni-variable analyses were performed to describe the association between HAPU risk factors and the occurrence of a day with HAPU. In the final step, multi-variable logistic regression models with Newey–West standard errors were constructed to estimate the odds of a HAPU Day. Exploratory analyses of nursing care costs were also performed.

Statistical analyses included descriptive statistics means, medians, minimum and maximum values, frequencies, and percentages. Categorical variables were compared using the chi-square test, while continuous variables without a normal distribution were analyzed using the Mann–Whitney U test and the Kruskal–Walli’s rank ANOVA with *post hoc* multiple comparisons of mean ranks for all groups.

Ward stays data represent a continuous time series with strong autocorrelation stemming from long patient hospitalizations and staff continuity. Because consecutive ICU care days are not independent, logistic regression models with Newey–West standard errors were used to estimate the odds of a HAPU Day. The HAC (Heteroskedasticity and Autocorrelation Consistent) approach applied here allows for the accurate estimation of robust standard errors and reliable *p*-values. Model specifications accounted for a mean length of stay of 21 days. The models were also adjusted for a categorical variable representing the observation year to control for long-term trends. Results are presented as adjusted odds ratios (aOR), model coefficients, 95% confidence intervals, and p-values.

The model-building strategy and variable selection were defined *a priori* based on the study objectives, clinical knowledge, organizational insights into ICU operations, and a review of current medical literature. All potential predictors and identified confounders (meaning length of stay of 21 days and observation year) were entered into the multivariable models simultaneously. Model goodness of fit and calibration were evaluated using the Akaike Information Criterion (AIC), Bayesian Information Criterion (BIC). Predictive performance was evaluated using receiver operating characteristic (ROC) analysis.

A significant level of 0.05 was adopted. Statistical analyses and graphical visualizations were performed using StataSE 18, STATISTICA 13, and Microsoft Excel.

### 2.10 Ethical considerations

The study was approved by the Bioethics Committee of the Medical University of Gdansk under no. NKBBN/573/2022 (2022/10/11).

## 3 Results

### 3.1 Year-by-year characteristics of HAPU days and study variables, 2019–2022

Over the four-year study period, HAPUs occurred in 8.1% of patients (86 cases), and HAPU Days accounted for 5.4% of all ICU Days (81 days). The proportion of HAPU Days decreased from 7.4% in 2019 to 4.1% in 2022. However, this trend was not statistically significant (*p* = 0.1612) (Table 2).

**Table 2:** Analysis of exposure variables associated with days with HAPU during the study period (2019–2022).

Variable	2019 (1)	2020 (2)	2021 (3)	2022 (4)	Total	*Post-hoc*
Days with HAPU						
*n* %	27 7.4	23 6.3	16 4.4	15 4.1	81 5.4	N/A
Chi-square test: *p* = 0.1612						
Patient-days						
Mean	7.8	7.9	7.8	7.8	7.9	N/A
Median	8.0	8.0	8.0	8.0	8.0	
Min–max	3.0–10.0	4.0–11.0	4.0–11.0	3.0–12.0	3.0–12.0	
Kruskal–Wallis test (overall): *p* = 0.0624						
NHPPD						
Mean	15.6	15.2	14.1	14.6	14.9	(1) vs. (3) *p* < 0.0001 (1) vs. (4) *p* < 0.0001 (2) vs. (3) *p* < 0.0001 (2) vs. (4) *p* < 0.0001
Median	15.0	15.0	13.5	13.3	13.7	
Min–max	10.7–36.0	10.5–30.0	9.0–27.0	8.4–34.5	8.4–36.0	
Kruskal–Wallis test (overall): *p* < 0.0001						
Risk of organ failure						
Mean	1.4	1.3	1.4	1.7	1.8	(1) vs. (3) *p* = 0.0013 (1) vs. (4) *p* < 0.0001 (2) vs. (3) *p* < 0.0001 (2) vs. (4) *p* < 0.0001
Median	1.0	1.0	2.0	2.0	2.0	
Min–max	0.0–5.0	0.0–5.0	0.0–6.0	0.0–5.0	0.0–5.0	
Kruskal–Wallis test (overall): *p* = 0.0149						
Single organ failure						
Mean	3.5	4.0	3.7	3.8	3.8	(1) vs (2) *p* = 0.0090
Median	4.0	4.0	4.0	4.0	4.0	
Min–max	0.0–7.0	0.0–8.0	0.0-8.0	0.0–10.0	0.0–10.0	
Kruskal–Wallis test (overall): *p* = 0.0149						
Two or more organ failures						
Mean	2.9	2.6	2.9	2.5	2.4	(1) vs. (3) *p* = 0.0003 (1) vs. (4) *p* < 0.0001
Median	3.0	3.0	2.0	2.0	2.0	
Min–max	0.0–7.0	0.0–6.0	0.0–7.0	0.0–6.0	0.0–6.0	
Kruskal–Wallis test (overall): *p* = 0.0001						

### 3.2 Demand for care: care complexity - comparison across the years 2019–2022

Patient care complexity shifted distinctly between 2019 and 2022 (Table 2), reflecting a changing ICU case-mix. Over time, the proportion of highly complex patients with multi-organ failure ( ≥2 organs) declined (*p* = 0.0149), whereas admissions of patients at risk of organ failure increased (*p* = 0.0001). Detailed data are presented in Supplementary Figure 2.

### 3.3 Educational structure of nurses - comparison across the years 2019–2022

Over the study period, the organizational profile of the nursing staff underwent a significant structural shift toward higher academic qualifications.

The proportion of nurses with university-level education systematically increased, accompanied by a corresponding decline in those with nurses responsible for general care.

Over the study period, the proportion of nurses with a master’s degree and specialist qualification increased (*p* < 0.001), as did the proportion of nurses with a master’s degree (*p* < 0.001). In contrast, the proportion of nurses with a bachelor’s degree decreased (*p* < 0.001), as did the proportion of nurses in the “other” category (*p* < 0.001).

From an operational standpoint, this upward trend in staff qualifications represents an enhanced capacity for evidence-based nursing care and standardized risk assessment on the ward (Table 3).

**Table 3:** Median distribution of nurses’ educational levels in 2019–2022 with Kruskal–Wallis post hoc testing.

2019 (1)	2020 (2)	2021 (3)	2022 (4)	Total	Box-and-whisker plot
Master of Science in Nursing (MSN) and specialization					
0%	10%	14%	23%	13%	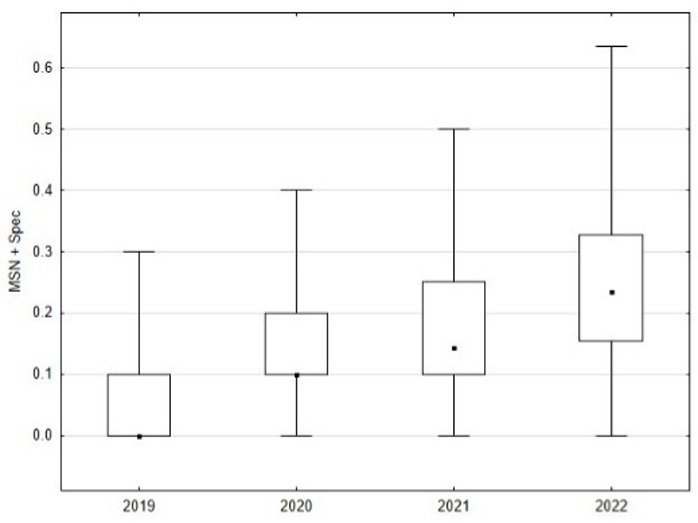
Kruskal–Wallis test (overall): *p* < 0.001 (1) vs. (2) *p* < 0.0001 (1) vs. (3) *p* < 0.0001 (1) vs. (4) *p* < 0.0001 (2) vs. (3) *p* < 0.0001 (2) vs. (4) *p* < 0.0001 (3) vs. (4) *p* < 0.0001					
Bachelor of Science in Nursing (BSN) and specialization					
20%	11%	20%	20%	20%	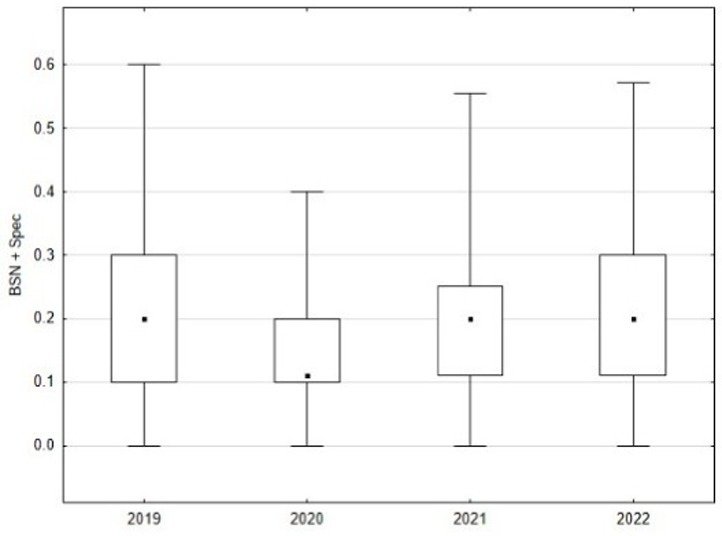
Kruskal–Wallis test (overall): *p* < 0.001 (1) vs. (2) *p* < 0.0001 (2) vs. (3) *p* < 0.0001 (2) vs. (4) *p* < 0.0001					
Master of Science in Nursing (MSN)					
10%	11%	13%	12%	10%	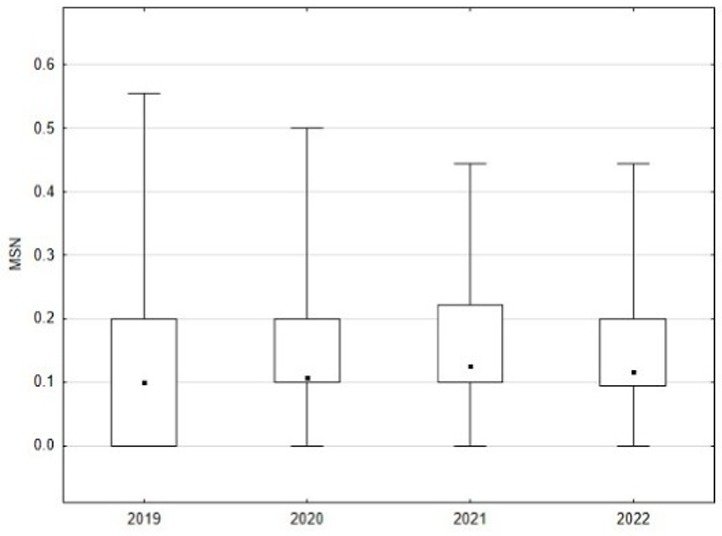
Kruskal–Wallis test (overall): *p* < 0.001 (1) vs. (2) *p* < 0.0001 (1) vs. (3) *p* < 0.0001 (1) vs. (4) *p* < 0.0001					
Bachelor of Science in Nursing (BSN)					
40%	30%	30%	23%	30%	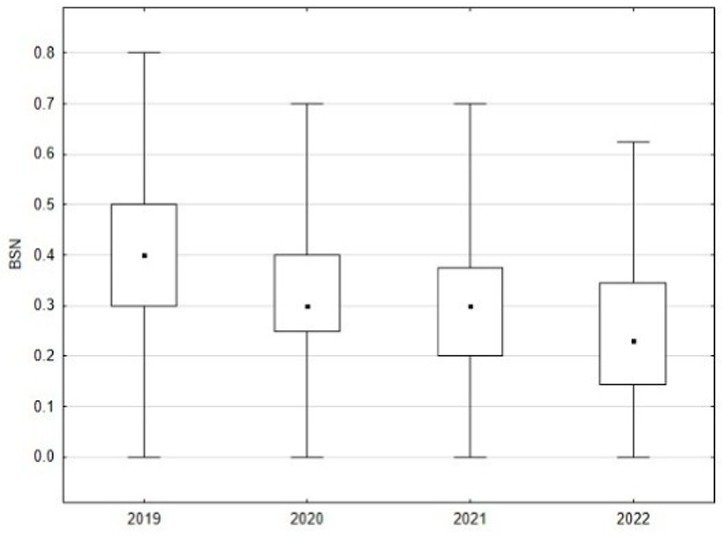
Kruskal–Wallis test (overall): *p* < 0.001 (1) vs. (2) *p* = 0.0298 (1) vs. (3) *p* < 0.0001 (1) vs. (4) *p* < 0.0001 (2) vs. (3) *p* < 0.0001 (2) vs. (4) *p* < 0.0001 (3) vs. (4) *p* < 0.0001					
Specialization					
0%	10%	0%	10%	5%	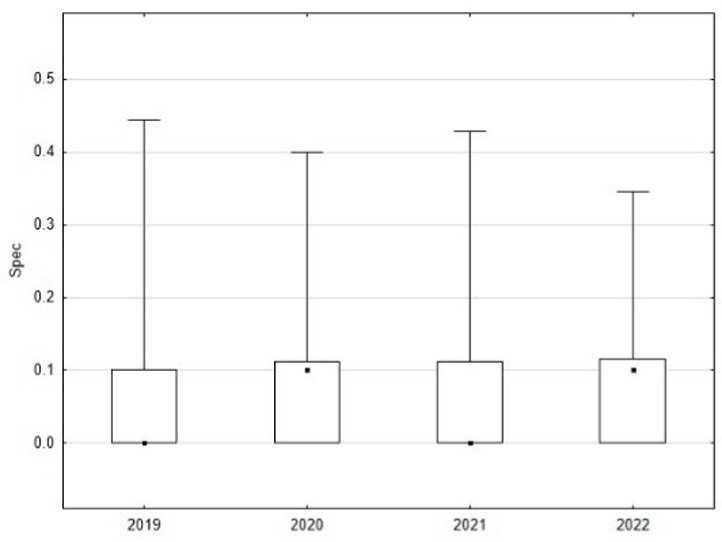
Kruskal–Wallis test (overall): *p* < 0.001 (1) vs. (2) *p* < 0.0001 (1) vs. (4) *p* < 0.0001 (1) vs. (3) *p* = 0.0020					
Other Registered Nurses					
20%	18%	13%	10%	15%	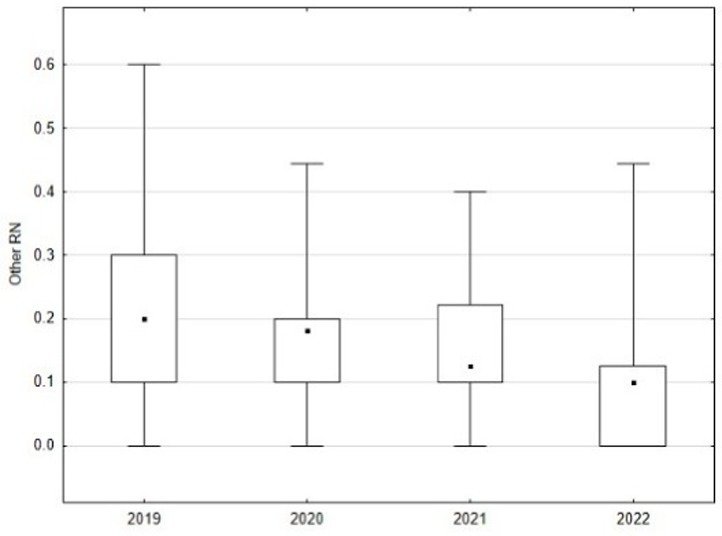
Kruskal–Wallis test (overall): *p* < 0.001 (1) vs. (2) *p* = 0.0002 (1) vs. (3) *p* = 0.0002 (1) vs. (4) *p* = 0.0002 (2) vs. (4) *p* < 0.0001 (3) vs. (4) *p* < 0.0001					

### 3.4 Workload: NHPPD and patient-days - comparison across the years 2019–2022

**Figure 2:**
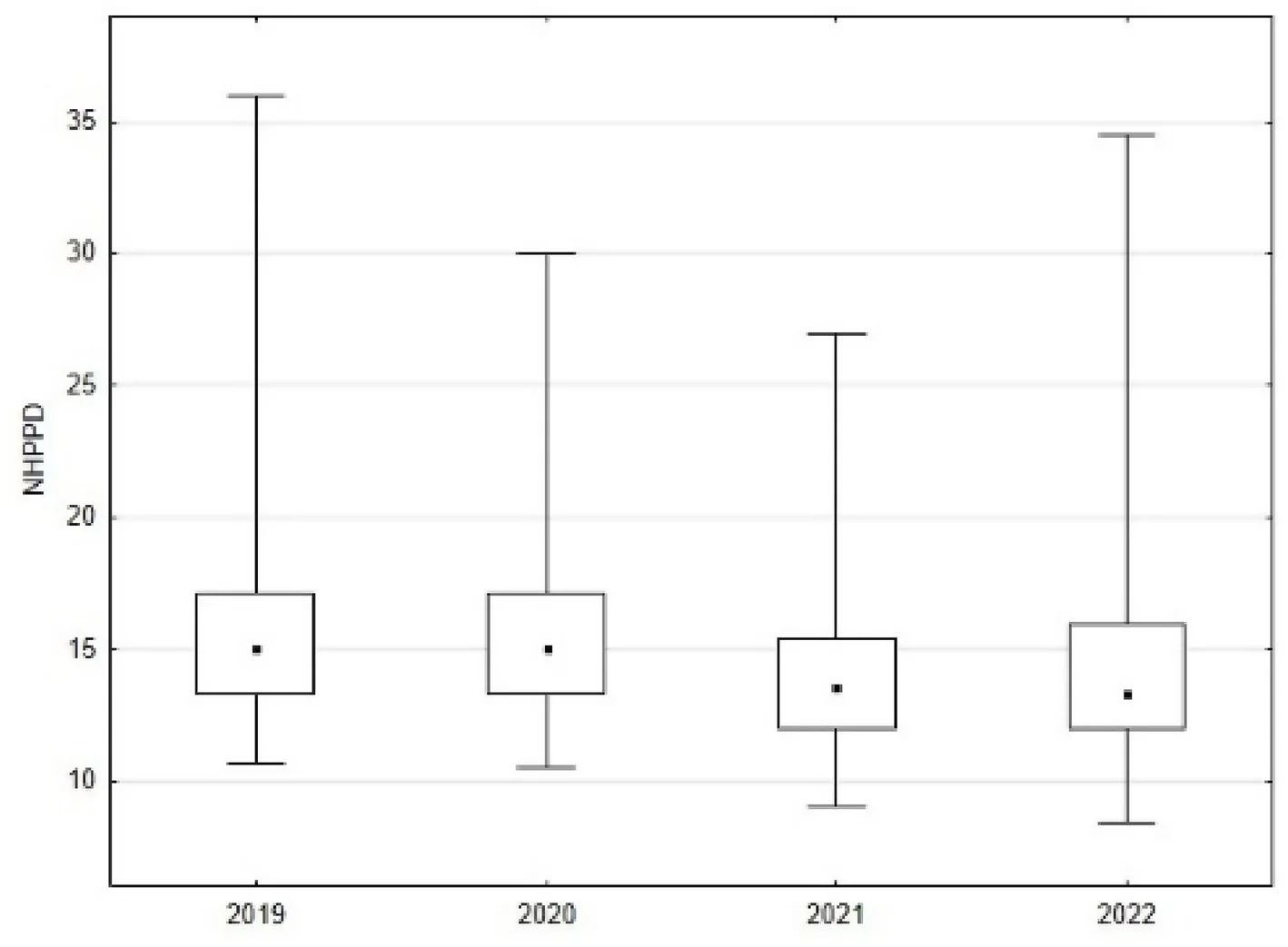
NHPPD characteristics across the study period.

While total patient-days remained stable across the study period (Me = 8), nursing staff availability for direct bedside care shifted. The NHPPD (Nursing Hours Per Patient Day) metric—calculated from a total of 166,456 care hours—demonstrated significant a decline over the study period (*p* < 0.001). From a workforce perspective, NHPPD dropped to its lowest point in 2021, directly capturing the heightened operational strain and reduced nurse-to-patient availability during the peak of the COVID-19 pandemic (Figure 2; Table 2).

### 3.5 Analysis of the impact of exposure factors on days with HAPU

Analysis HAPU-affected days showed no significant differences regarding NHPPD, nursing educational background, or the degree of patient organ failure (Table 4).

**Table 4:** Workload, education, and demand for care in relation to days with and without HAPU.

Variable	HAPU-days cohort	Days without HAPU cohort	Mann–Whitney U test
NHPPD			
Mean	14.3	14.9	*p* = 0.2068
Median	13.3	13.7	
Min-max	10.8–25.9	8.4–36.0	
Patient-days (2019-2022)			
Mean	8.2	7.9	*p* = 0.0331
Mean rank-sum	826	725	
Median	8.0	8.0	
Min-max	4.0–10.0	3.0–12.0	
Patient-days 2019			
Mean	8.5	7.8	*p =* 0.0027
Median	9.0	8.0	
Min-mix	6.0–10.0	3.0–12.0	
Nurse education MSN + Spec (A)			
Mean	14%	15%	*p* = 0.4198
Median	10%	13%	
Min-max	0–50%	0–63%	
Nurse education BSN + Spec (B)			
Mean	20%	18%	*p* = 0.2202
Median	20%	20%	
Min-max	0–50%	0–60%	
Nurse education - MSN (C)			
Mean	13%	13%	*p* = 0.4346
Median	10%	11%	
Min-max	0–40%	0–56%	
Nurse education – BSN (D)			
Mean	29%	31%	*p* = 0.2651
Median	30%	30%	
Min-max	0–60%	0–80%	
Nurse education – Spec (E)			
Mean	8%	7%	*p* = 0.5436
Median	10%	10%	
Min-max	0–43%	0–44%	
Nurse education – Others RN (F)			
Mean	16%	15%	*p* = 0.8932
Median	13%	13%	
Min-max	0–60%	0–50%	
Risk of organ failure			
Mean	1.6	1.6	*p* = 0.7607
Median	2.0	1.0	
Min-max	0–4	0–6	
Single organ failure			
Mean	4.0	3.7	*p* = 0.1442
Median	4.0	4.0	
Min-max	1–8	0–10	
≥2 organ failures			
Mean	2.6	2.6	*p* = 0.9552
Median	2.0	3.0	
Min-max	0–6	0–7	

From an operational perspective, care complexity itself was not a primary driver of bed-level HAPU risk. Rates of HAPU-affected days remained stable regardless of organ failure severity—showing no significant differences among patients at risk (*p* = 0.7607), with single organ failure (*p* = 0.1442), or with multi-organ failure (*p* = 0.9552). This suggests that HAPU exposure on the ward depends more on overarching care processes and exposure time than on baseline organ instability alone.

In contrast, total patient volume emerged as a notable operational factor, with HAPU events occurring more frequently during periods of higher bed occupancy (Figure 2). However, year-stratified analysis revealed that this volume-driven risk was confined solely to 2019, where days with HAPU coincided with a higher median patient count than HAPU-free days (9.0 vs. 8.0 patients, *p =* 0.0027). In subsequent years (2020–2022), this association dissipated, indicating that HAPU risk became decoupled from daily ward occupancy levels over time.

### 3.6 Logistic regression models with Newey–West standard errors for days with HAPU

The simultaneous associations between multiple organizational exposure variables and HAPU Day occurrence were assessed using multivariable logistic regression models with Newey–West robust standard errors. Daily ICU observations constituted a time series with serial correlation stemming from prolonged patient stays and recurring staffing patterns. Accordingly, Newey–West standard errors were estimated using a lag length corresponding to the average ICU length of stay (21 days). All models were further adjusted for the year of hospitalization (2019–2022) to account for long-term trend over the study period.

Model 1 evaluated the relationship between nursing availability (NHPPD) and HAPU Day occurrence (Table 5). Although NHPPD was not statistically significant in univariable comparisons, the multivariable model demonstrated that each additional hour of NHPPD was associated with a 9% reduction in the odds of a HAPU Day (aOR = 0.91; 95% CI: 0.83–0.99; *p* = 0.035).

**Table 5:** Logistic regression models with Newey–West standard errors.

Variable	Coef.	aOR	95% CI	*p*
Model 1 – association between days with HAPU and NHPPD				
NHPPD	–0.099	0.906	0.827–0.993	0.035
2019	Base category			
2020	–0.218	0.804	0.471–1.374	0.425
2021	–0.705	0.494	0.238–1.024	0.058
2022	– 0.745	0.475	0.210–1.073	0.073
Model 2 – association between days with HAPU and patient-days				
Patient-days	0.171	1.187	1.003–1.405	0.047
2019	Base category			
2020	–0.195	0.823	0.481–1.407	0.476
2021	–0.587	0.556	0.275–1.124	0.102
2022	–0.680	0.507	0.228–1.125	0.095

Dependent variable: Days with HAPU: Model 1 – Association between days with HAPU and NHPPD; Model 2 – Association between days with HAPU and patient-days.

Model 2 assessed the impact of daily patient volume (Table 5). In this model, each additional patient-day was associated with a 19% increase in the odds of a HAPU Day (aOR = 1.19; 95% CI: 1.00–1.40; *p* = 0.047).

The ROC curves representing the models showed a moderate predictive ability. The area under the curve (AUC) was 0.602 (95% CI: 0.574-0.625) for Model 1 and 0.600 (95% CI: 0.576-0.627) for Model 2. The models showed limited discriminatory performance - Supplementary Figures 3A–C.

After accounting for long-term autocorrelation and long-term trends, the multivariable models demonstrated that organizational structural factors—specifically nursing availability (NHPPD) and daily patient volume—were significantly associated with the odds of HAPU Day occurrence. However, the models exhibited relatively modest discriminatory power, indicating that key determinants of HAPU risk were not captured within the available administrative dataset. In the context of hospital-acquired pressure injury prevention, such unmeasured factors typically encompass granular patient-level clinical profiles and acute physiological risk factors, which fell outside the scope of this organizational-level investigation.

Model goodness-of-fit for the generalized linear models was evaluated using the Akaike Information Criterion (AIC) and Bayesian Information Criterion (BIC). Model 1 yielded an AIC of 625.9 and a BIC of 652.4, whereas Model 2 yielded an AIC of 626.5 and a BIC of 653.0. Because the inter-model differences for both information criteria were less than 1 point (ΔAIC < 1, ΔBIC <1), neither model demonstrated statistical superiority in fit, indicating comparable explanatory value between nursing hours and daily patient workload.

**Figure 3:**
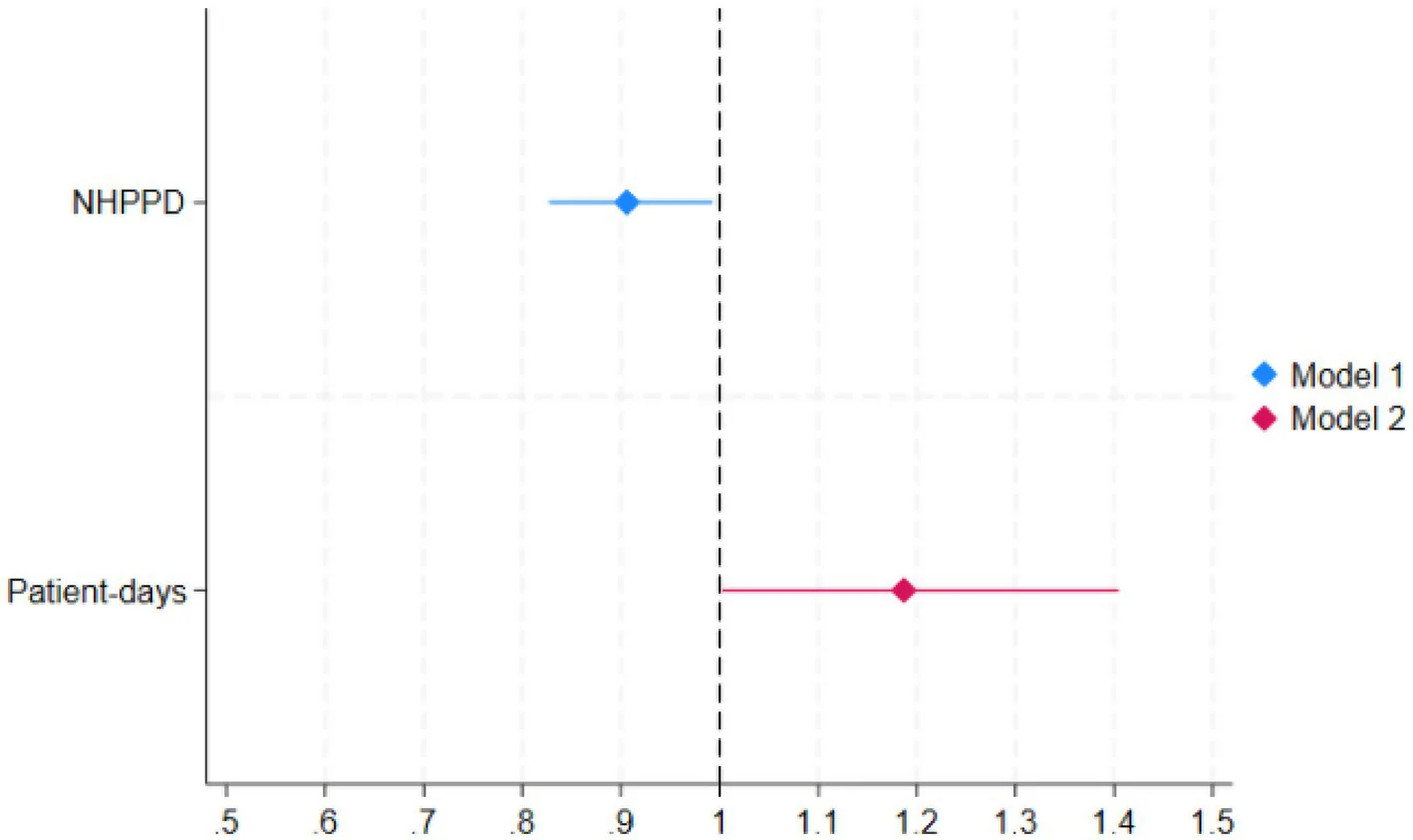
Forest plot of adjusted odds ratios (aORs) and 95% confidence intervals for HAPU Day occurrence.

The adjusted odds ratios (aOR) and their corresponding 95% confidence intervals for the organizational exposure variables are visually summarized in the forest plot (Figure 3).

### 3.7 Nursing care costs associated with Hapu days

A comparative economic analysis of nursing salary costs per patient-day revealed no statistically significant difference between HAPU-affected days and non-HAPU days (mean: 1,958.33 EUR, median: 1,943.80 EUR vs. mean: 2,074.50 EUR, median: 2,039.28 EUR; Mann–Whitney U test with continuity correction, *p* = 0.07 Table 6).

**Table 6:** Nursing care costs per day across study years and on HAPU and non-HAPU days.

Variable	Nursing care costs per day 2019-2022 (PLN and EUR)									Average nursing care costs per day on Non-HAPU and HAPU days (PLN and EUR)		
		2019		2020		2021		2022		Non-HAPU days		HAPU days
Mean	8,602.7 2,001.6	PLN EUR	8,457.3 1,903.0	PLN EUR	9,342.8 2,041.3	PLN EUR	10,857.9 2,317.1	PLN EUR	9,338.1 2,074.5	PLN EUR	8,914.7 1,958.3	PLN EUR
Median	8,848.6 2,058.8	PLN EUR	8,252.2 1,856.8	PLN EUR	9,093.0 1,986.7	PLN EUR	11,432.3 2,439.6	PLN EUR	9,179.5 2,039.3	PLN EUR	8,848.6 1,943.8	PLN EUR
Min	5,226.0 1,215.9	PLN EUR	4,861.7 1,093.9	PLN EUR	5,679.9 1,241.0	PLN EUR	6,629.2 1,414.6	PLN EUR	4,861.7 1,080.1	PLN EUR	5,679.9 1,247.7	PLN EUR
Max	10,887.5 2,533.2	PLN EUR	12,743.6 2,867.4	PLN EUR	12,920.6 2,822.9	PLN EUR	15,367.1 3,279.3	PLN EUR	15,367.1 3,413.9	PLN EUR	12,920.6 2,838.3	PLN EUR
*p*-value	Kruskal-Walli’s test: *p* < 0.001									Mann–Whitney test: *p* = 0.07		

The lowest median cost was recorded in 2020 (1,856.79 EUR), followed by 2021 (1,986.67 EUR) and 2019 (2,058.76 EUR), whereas the highest median cost occurred in 2022 (2,439.60 EUR). *Post hoc* pairwise comparisons demonstrated significant cost differences between nearly all year pairs (2019 vs. 2021, 2019 vs. 2022, 2020 vs. 2021, 2020 vs. 2022, and 2021 vs. 2022; *p* < 0.001).

**Figure 4:**
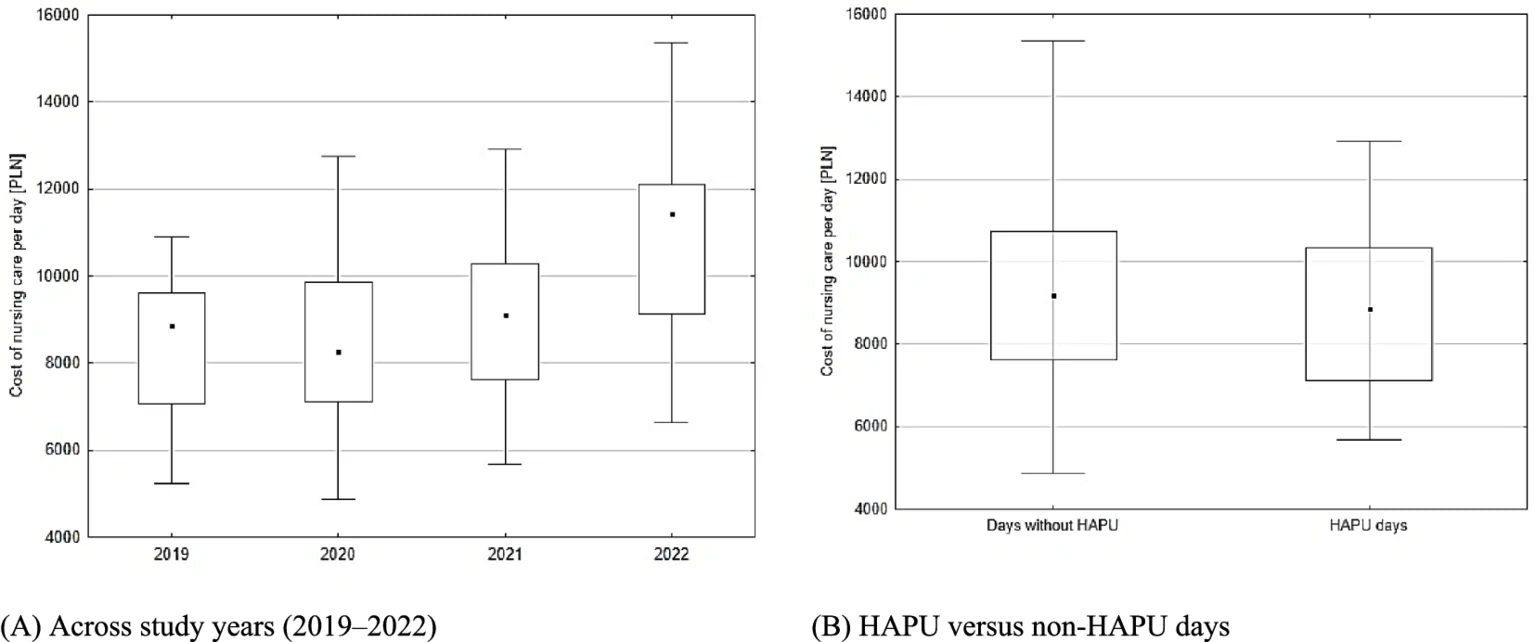
Distribution of nursing care costs per day according to study year and HAPU occurrence. **(A)** Across study years (2019–2022); **(B)** HAPU versus non-HAPU days.

Figures 4A,B present the distribution of nursing care costs per day across study years and according to HAPU occurrence.

## 4 Discussion

The present study examined the associations between nursing workload, staffing qualification structure, nursing care costs, and the occurrence of hospital-acquired pressure ulcer (HAPU) Days in an intensive care unit using routinely collected retrospective organizational data. The principal finding was that HAPU Days were characterized by a higher number of patient-days and lower Nursing Hours per Patient Day (NHPPD) compared with non-HAPU Days, whereas the educational structure of the nursing workforce and care complexity were not significantly associated with HAPU Day occurrence. These findings support the importance of organizational conditions in HAPU prevention and are consistent with previous studies reporting associations between nursing workload, staffing availability, and adverse patient outcomes, including pressure ulcers (37, 58, 59). In Poland, nurse staffing in ICUs is regulated by national standards based on minimum staffing requirements (52), whereas contemporary international recommendations increasingly advocate flexible staffing models that adjust nursing resources according to patients’ changing care needs and workload (60). By analyzing routinely collected ICU-day data rather than individual hospitalization episodes, the present study provides evidence on how daily organizational conditions are associated with HAPU occurrence. To our knowledge, this is one of the first Polish studies to evaluate these relationships using objective indicators derived from hospital information systems, including NHPPD and patient-days.

### 4.1 Days with HAPU and the number of patient-days

The multivariable logistic regression model with Newey–West standard errors identified the number of patient-days as a significant organizational factor associated with HAPU Days. Each additional patient-day was associated with a 19% increase in the odds of a HAPU Day, indicating that fluctuations in daily patient occupancy may influence the occurrence of pressure ulcers at the unit level. This finding is consistent with previous ICU studies reporting that increasing patient volume is associated with higher nursing workload and a greater risk of adverse patient outcomes (61, 62). Unlike nurse-to-patient ratios, the number of patient-days reflects the overall demand for nursing care within the unit and captures variations in workload resulting from changing patient occupancy. Higher patient occupancy may reduce the time available for preventive nursing activities, particularly when staffing resources do not increase proportionally to patient demand.

Although the present study did not directly assess nursing care processes, the observed association supports the hypothesis that organizational workload indicators should be considered when planning ICU staffing. These findings reinforce the importance of monitoring patient occupancy together with NHPPD rather than relying solely on minimum staffing requirements. Future multicentre longitudinal studies should examine whether dynamic staffing models that incorporate daily patient occupancy can contribute to reducing the occurrence of HAPU Days across different ICU settings (63–65).

### 4.2 Days with HAPU and NHPPD

In the present study, NHPPD did not differ significantly between HAPU and non-HAPU Days in the univariable analyses. However, after adjustment for and year of hospitalization, higher NHPPD was independently associated with lower odds of HAPU Day occurrence (OR = 0.91; 95% CI: 0.83–1.00). This finding suggests that the relationship between nursing workload and HAPU occurrence becomes evident only after accounting for other confounding factors related to the year of hospitalization, which were not analyzed in this study. Similar associations between higher nursing staffing levels and improved patient outcomes have been reported in previous ICU studies (37, 66). NHPPD represents an objective measure of nursing resource availability and reflects the balance between available nursing hours and patient care demand. Unlike fixed nurse-to-patient ratios, NHPPD is sensitive to daily changes in workload and therefore may better characterize organizational conditions in intensive care units. These findings support the inclusion of workload indicators such as NHPPD in staffing evaluation and quality monitoring, although additional studies are required to determine optimal staffing thresholds for different ICU settings.

Compared with staffing approaches reported in other healthcare systems, the workload category used in the present study reflects a more dynamic assessment of nursing resource availability. In many countries, ICU staffing is increasingly based not only on fixed nurse-to-patient ratios, but also on patient acuity, care complexity, skill mix, and real-time workload indicators (67, 68). In contrast, Polish ICU staffing regulations remain largely based on formal nurse-to-bed ratios, which may not fully capture day-to-day variation in patient demand (50).

Although NHPPD and patient-days were significantly associated with HAPU occurrence, the models demonstrated only limited discriminatory ability. This indicates that organizational variables alone are insufficient to accurately predict HAPU occurrence. The findings suggest that additional patient-level factors—including age, comorbidities, nutritional status, severity of illness, organ dysfunction, and pressure ulcer risk—are likely to play an important role.

Consequently, the present models should be regarded as exploratory organizational models rather than clinical prediction tools. Future multicenter studies integrating organizational and patient-level variables are needed to improve predictive performance.

### 4.3 Days with HAPU and care complexity

A novel aspect of the present study was the inclusion of care complexity represented by TISS-28-based organ failure burden. Unlike many previous studies focusing primarily on staffing indicators (69), the present analysis incorporated organ failure burden as an organizational indicator of nursing care demand. This approach reflects the clinical reality of intensive care, where nursing workload depends not only on the number of patients but also on the intensity and complexity of care required. In Poland, TISS-28 is routinely used to support ICU staffing planning according to patient acuity and organ failure burden (50, 57).

During the study period, changes in patient complexity were observed, particularly following the COVID-19 pandemic, when the proportion of patients with two or more organ failures decreased, whereas the number of patients at risk of organ failure increased. Similar changes in ICU case-mix have been described internationally after the pandemic (70–72). Although organ failure burden was not significantly associated with HAPU Day occurrence, its inclusion enabled a more comprehensive assessment of organizational workload than staffing indicators alone. The absence of a statistically significant association may indicate that the TISS-28 categories used in the present study do not fully capture the patient-level factors directly related to pressure ulcer development. Alternatively, organizational factors such as NHPPD and daily patient occupancy may explain part of the variation in HAPU Days independently of care complexity. These findings suggest that organizational workload should be interpreted as a multidimensional concept that incorporates both staffing resources and patient care demand.

Consequently, staffing models based exclusively on fixed nurse-to-bed ratios may not adequately reflect daily fluctuations in care demand. Integrating workload indicators such as NHPPD with measures of patient complexity may provide a more comprehensive framework for organizational planning intensive care. Future multicenter studies should evaluate whether more detailed patient-level severity measures improve the assessment of organizational workload and HAPU risk.

### 4.4 Days with HAPU and nurses’ education

One of the key findings of this study was the lack of a statistically significant association between nurses’ educational levels and the occurrence of HAPU days, despite changes in the educational structure of the ICU nursing team over time. The lack of a statistically significant association between nurses’ educational structure and HAPU days should be interpreted as a null finding rather than as evidence that education or professional competencies are unrelated to HAPU prevention. This result may reflect limited statistical power, the single-center design, limited variability in the educational profile of the ICU nursing team, and the stronger influence of workload-related factors such as NHPPD and patient-days. The median proportion of nurses holding a bachelor’s degree was approximately 30%, while nurses with a master’s degree represented 10–11% of the team. The median proportion of nurses with specialist qualifications was 40% in both HAPU and non-HAPU cohorts. Although the proportion of nurses holding university degrees alone remained below the 50% threshold recommended in national ICU staffing standard (73) and lower than proportions reported in some internationally (74). Specifically, when academic degrees were evaluated alongside postgraduate specialist qualifications, the overall proportion of nursing staff with advanced clinical competencies approached the recommended 50% benchmark (73).

Previous studies have suggested that higher nursing education levels are associated with improved patient outcomes and greater implementation of evidence-based nursing practices (18, 75, 76). However, this relationship was not observed in the present study with respect to HAPU days. The association between nursing qualifications and HAPU occurrence is inherently multifactorial, depending heavily on practical clinical experience and hands-on competency in executing evidence-based prevention strategies (77, 78).

The absence of a statistically significant association between nurses’ educational qualifications and HAPU occurrence should be interpreted within the context of both the Polish nursing education framework and the organizational characteristics of the study setting. Although educational structure was not associated with HAPU days in the univariable comparisons, several key mechanisms likely explain this finding.

Professional experience was not assessed directly as a standalone variable. However, in Poland, entry into an anesthesiology and intensive care nursing specialization requires a minimum of 2 years of clinical practice, and a Master’s degree involves an additional 2 years of graduate education. Consequently, nurses holding specialist qualifications or Master’s degrees possess substantial baseline clinical exposure, creating a relatively homogeneous competency profile across the ICU team that may have limited the statistical variability needed to detect education-specific effects.

All nursing staff operated within a standardized 12-h shift system (07:00–19:00 and 19:00–07:00) and adhered to uniform pressure injury prevention protocols in an accredited hospital. These protocols included systematic risk assessment, routine skin inspection, structured documentation, EPUAP-compliant injury classification, and verification of all HAPU cases by a dedicated DECUBITUS nurse coordinator. Such standardized clinical pathways minimize practice variability and reduce disparities attributable solely to formal academic credentials (79). These findings align with international literature indicating that the effect of nursing education on nursing-sensitive outcomes is strongly modified by organizational context—including staffing adequacy, work environment, safety culture, and protocol adherence (13, 28, 31, 80–83).

While workload metrics (NHPPD and patient-days) demonstrated significant associations in our logistic regression models (Newey–West correction), this does not imply that structural workload outweighs educational preparation. ICU care fundamentally relies on team coordination and practical execution (84). Therefore, the lack of an independent association for education should not be misconstrued as evidence that formal qualifications or advanced competencies are unimportant for patient safety.

Certain organizational determinants, such as nurse turnover, absenteeism, and staff replacement rates, were unavailable in the dataset and could not be evaluated (16, 78, 85–88). Given these limitations and the single-center sample size, these findings should be interpreted cautiously. Future prospective, multicenter longitudinal studies integrating nursing education, clinical experience, turnover, work organization, and patient-level clinical variables are needed to fully elucidate the joint impact of workforce competencies and organizational dynamics on HAPU risk in intensive care settings.

### 4.5 Nursing care costs associated with HAPU days

The cost analysis is exploratory in nature. The present study demonstrated that nursing care costs per patient-day were lower on days with HAPU than on days without HAPU. Mean and median nursing care costs were lower by approximately 4–5% in HAPU days. However, this difference did not reach statistical significance; therefore, the cost-related findings should be interpreted cautiously and as hypothesis-generating rather than confirmatory.

These findings should not be interpreted as evidence that omitted nursing care directly caused HAPU occurrence. Hospital-acquired pressure ulcers are multifactorial adverse events influenced by patient-related, clinical, and organizational factors, including age, comorbidities, immobility, nutritional status, severity of illness, organ failure burden, case-mix, and the COVID-19 pandemic (89–92). Because the present study did not directly measure missed nursing care, individual preventive interventions, or patient-level nursing activities, nursing care costs cannot be considered a direct proxy for the quality or completeness of bedside nursing care.

Instead, nursing care costs should be interpreted as an organizational indicator reflecting nursing workforce resources (31, 37, 53, 93, 94). In the present study, they were derived from aggregated labor costs and were influenced by staffing levels, qualification structure, salary scales, overtime, and the professional experience of the nursing workforce. Consequently, differences in nursing care costs may reflect differences in workforce composition and resource availability rather than differences in the quality of nursing care delivered.

Although no statistically significant differences in the educational structure were observed between HAPU and non-HAPU days, descriptive differences in the proportion of nurses with advanced qualifications were noted. At the same time, the number of patient-days was significantly higher in the HAPU group, indicating greater workload and organizational pressure during these periods. In this context, the cost-related findings may contribute to the scientific literature by demonstrating how routinely collected payroll and staffing data can be used as indirect organizational indicators of nursing workforce availability, skill mix, and workload pressure (28, 31, 81, 82). However, these findings should be interpreted as exploratory and require confirmation in longitudinal multicenter studies incorporating direct measures of missed nursing care, preventive nursing interventions, and patient-level clinical risk factors.

Therefore, the observed descriptive difference in nursing care costs between lower nursing care costs and HAPU days should be regarded as a hypothesis generated by this study rather than evidence of a causal mechanism. The findings suggest that nursing care cost data may help characterize the organizational context in which HAPU days occur; however, they do not establish that differences in nursing resources caused HAPU occurrence.

### 4.6 Exploratory performance of the organizational models

Although NHPPD and patient-days were significantly associated with HAPU Day occurrence, the regression models demonstrated limited discriminatory performance. This finding must be interpreted in light of the macro-level organizational intent of these analyses. The models intentionally incorporated operational variables routinely monitored for ICU staffing and workload management in Poland—such as NHPPD, patient-days, and TISS-28-derived organ failure burden—rather than a granular battery of patient-level clinical predictors. Consequently, they were designed to evaluate system-level structural associations rather than function as bedside clinical HAPU risk-prediction tools.

Statistical significance and discriminatory power address fundamentally different analytical questions: the former determines whether an organizational factor is reliably associated with the outcome, whereas the latter reflects how accurately a model distinguishes between ICU days with and without HAPU events.

The relatively modest AUC values indicate that administrative and staffing variables alone are insufficient to discriminate between HAPU and HAPU-free days with high sensitivity and specificity. This limited discrimination does not negate the statistically significant effects observed for NHPPD and patient-days; rather, it underscores the multifactorial etiology of pressure ulcer development. It highlights the boundary conditions of models relying solely on aggregated staffing, workload, and care-demand metrics. Other critical unmeasured drivers likely include patient-specific parameters such as age, comorbidities, immobility, impaired tissue perfusion, nutritional deficits, illness severity, vasopressor administration, mechanical ventilation, surgical case-mix, and validated pressure ulcer risk scores.

Future research should integrate system-level organizational indicators with patient-level clinical characteristics and direct process measures of preventive care to improve model discrimination and deliver a more comprehensive evaluation of HAPU risk.

Although missed nursing care has been proposed as a mechanism linking nursing workload with adverse patient outcomes, including HAPUs in ICUs (59, 95, 96), the present study did not directly measure missed nursing care, omitted preventive interventions, or other nursing care processes. Therefore, no causal inferences regarding this mechanism can be drawn from the present findings, and the concept should be interpreted only as a plausible explanatory pathwaythe present study did not directly measure nursing care processes.

## 5 Summary

The findings of this study indicate that safe ICU nurse staffing should be informed not only by fixed nurse-to-bed ratios but also by dynamic indicators of workload and care demand, including NHPPD, daily patient volume, and organ failure complexity. During periods of severe organizational strain, such as the COVID-19 pandemic, static staffing models prove insufficient to capture daily fluctuations in patient acuity and nursing workload. Consequently, future workforce strategies should leverage routinely collected hospital administrative data—such as payroll records, nursing-sensitive outcomes, and clinical complexity metrics—to enable more responsive, evidence-informed staffing planning.

In this context, mathematical modeling and advanced knowledge management frameworks offer strong potential to support safer staffing decisions by integrating real-world data on workload, workforce composition, and adverse events. Importantly, because the present study did not directly evaluate artificial intelligence-based decision-support tools, references to AI and predictive automation should be understood strictly as future horizons for research and workforce infrastructure, rather than direct empirical conclusions from these findings.

## 6 Conclusion

Mathematical modeling was applied to estimate the odds of HAPU day occurrence, which was 5.4% in the evaluated ICU—a figure comparable to rates reported in other hospital settings. Higher NHPPD showed a protective association with HAPU days, whereas an increased daily patient census was associated with elevated risk. Specifically, each additional hour of NHPPD was associated with a 9% reduction in the odds of HAPU day occurrence (aOR = 0.91), while each additional patient per day increased the odds by 19% (aOR = 1.19).

These findings indicate that operational workload indicators—namely NHPPD and daily bed occupancy—are key contextual factors associated with HAPU events in intensive care settings. Notably, although the proportion of nurses with university degrees systematically increased over time, formal educational level was not independently associated with HAPU occurrence in this dataset.

Given the retrospective, single-center cohort design, the findings should be interpreted cautiously and warrant further validation in prospective multicentre longitudinal studies. The cost analysis was exploratory and should be interpreted as hypothesis-generating.

## Data Availability

The raw data supporting the conclusions of this article will be made available by the authors, without undue reservation.
