# Supplementary material for: Nursing workload, staffing qualifications, and nursing care costs associated with hospital-acquired pressure ulcer days in the ICU: a retrospective cohort study
Source: Front Public Health. 2026 Sep 9;14:1836283. doi: 10.3389/fpubh.2026.1836283 (PMC13597870; doi:10.3389/fpubh.2026.1836283)
Supplement: Supplementary file 1 [file Supplementary_file_1.docx]

Supplementary Material

# 1 Supplementary Figures and Tables

## 1.1 Supplementary Tables

**Supplementary Table ST1. Classification of patients according to intensive care requirements**

| Category I | 0–19 points | Patients physiologically stable and requiring prophylactic observation. |
| --- | --- | --- |
| Category II | 20–34 points | Patients physiologically stable but requiring intensive nursing care and continuous monitoring. |
| Category III | 35–60 points | Hemodynamically severe and unstable patients. |
| Category IV | >60 points | Patients with indication for compulsory admission to the ICU with continuous and specialised medical and nursing assistance. |

**Supplementary Table ST2 – Study-specific classification of organ failure based on selected TISS-28 criteria**

| System | Criteria | Points | Interpretation |
| --- | --- | --- | --- |
| Respiratory system | The patient required mechanical ventilation or high-flow oxygen therapy, including CPAP; | 5 | A total score of ≥5 points qualified the patient as having respiratory system failure. |
| Cardiovascular system | The patient required pharmacological (e.g., inotropic drugs) or mechanical support and had a central venous catheter; | 4: multiple vasoactive drugs (vasopressors: epinephrine, norepinephrine, dopamine), inodilators: amrinone, dobutamine and vasodilators: nitroglycerin, sodium nitroprusside (drugs administered orally, via feeding tube, or subcutaneously were not included);  2: central venous catheter | A total score of ≥6 points qualified the patient as having cardiovascular system failure. |
| Renal system | The patient required renal replacement therapy, such as continuous renal replacement therapy; | 3: hemofiltration, dialysis  2: required urine output monitoring every 8 hours | A total score of ≥5 points qualified the patient as having renal system failure. |
| Nervous system | The patients were assessed based on level of consciousness and neurological function, most often following central nervous system injury; | 4: intracranial pressure (ICP) monitoring was required; | A score of 4 points qualified the patient as having central nervous system failure. |
| Gastrointestinal system | The patient had undergone surgery involving the abdominal cavity or gastrointestinal tract and required parenteral nutrition or administration of energy substrates and amino acids via a central venous catheter; | 3: parenteral nutrition | A score of 3 points qualified the patient as having gastrointestinal system failure. |
| Musculoskeletal system | The patient had sustained trauma that was not surgically managed and posed an immediate life-threatening condition (e.g., multiple long-bone fractures, pelvic fractures, spinal fractures with damage or high risk of spinal cord injury). | 1: frequent dressing changes were assigned;  3: drain care | A total score of ≥4 points qualified the patient as having musculoskeletal system failure. |

Legend: https://sfar.org/scores2/tiss2.php

The cohort comprised 1,056 patients hospitalised between 2019 and 2022. Patients in the cohort of days with HAPU had a mean age of 62 years (approximately one year higher than those without HAPU), a lower proportion of patients aged >65 years (58% vs. 62%), and a longer length of hospital stay by 18 days compared with the cohort without days with HAPU (38 vs. 20 days). In both cohorts, the predominant ICD-10 diagnosis was respiratory failure. The percentages of the most common causes of hospitalisation are presented in Table S3.

**Supplementary Table ST3. Characteristics of patients hospitalised in the period 2019–2022 (N = 1,056)**

| Patient profile | HAPU cohort | HAPU-free cohort |
| --- | --- | --- |
| Number of patients | 86 (8%) | 970 (92%) |
| Number of men | 58 (67%) | 597 (62%) |
| Age | 62 | 61 |
| Number of patients aged ≥65 years | 650 (58%) | 50 (62%) |
| Length of hospital stay (days) | 38 | 20 |
| Most frequent ICD-10 diagnosis | J96 (40%) | J96(11%) |
| Second most common ICD-10 diagnosis | I46 (15%) | S06 (10%) |
| Third most common ICD-10 diagnosis | S06 (10%) | A41 (7%) |

## 1.2 Supplementary Figures

**Supplementary Figure SF1. Conceptual framework of organisational factors examined in relation to HAPU Day occurrence.**

**Supplementary Figure SF2.** **Two or more organ failures in 2019–2022; Number of patients with one organ failure in 2019–2022; Number of patients at risk of organ failure in 2019–2022.**

**Supplementary Figure SF3.**

| YEAR 1  **Figure 3A. Probability model of days with HAPU according to NHPPD.** | |
| --- | --- |
|  |  |
| **Fig. 3B. Receiver operating characteristic (ROC) curve for Model 1 predicting days with HAPU.** | **Fig. 3C. Receiver operating characteristic (ROC) curve for Model 2 predicting days with HAPU.** |

Association between NHPPD and the probability of HAPU Day occurrence

Probability of HAPU occurrence

| NHPPD | rok1 | rok2 | rok3 | rok4 |
| --- | --- | --- | --- | --- |
| 8 | 14.1268% | 11.683% | 7.5181% | 7.24399% |
| 9 | 12.9724% | 10.7034% | 6.86062% | 6.60877% |
| 10 | 11.8993% | 9.79688% | 6.25674% | 6.02563% |
| 11 | 10.9039% | 8.95942% | 5.70277% | 5.49092% |
| 12 | 9.98224% | 8.18705% | 5.19513% | 5.00114% |
| 13 | 9.13053% | 7.47579% | 4.7304% | 4.55293% |
| 14 | 8.34476% | 6.82174% | 4.30537% | 4.14315% |
| 15 | 7.62094% | 6.22106% | 3.91695% | 3.76879% |
| 16 | 6.95515% | 5.67005% | 3.56227% | 3.42705% |
| 17 | 6.34352% | 5.16516% | 3.23862% | 3.11529% |
| 18 | 5.78234% | 4.70299% | 2.94348% | 2.83106% |
| 19 | 5.26801% | 4.2803% | 2.6745% | 2.57207% |
| 20 | 4.79709% | 3.89406% | 2.42948% | 2.33621% |
| 21 | 4.36634% | 3.54137% | 2.20639% | 2.12151% |
| 22 | 3.97265% | 3.21956% | 2.00337% | 1.92615% |
| 23 | 3.61311% | 2.92611% | 1.81869% | 1.74845% |
| 24 | 3.28501% | 2.65866% | 1.65074% | 1.58689% |
| 25 | 2.98577% | 2.41506% | 1.49807% | 1.44003% |
| 26 | 2.71303% | 2.19327% | 1.35932% | 1.30659% |
| 27 | 2.46457% | 1.99143% | 1.23326% | 1.18536% |
| 28 | 2.23834% | 1.80783% | 1.11876% | 1.07526% |
| 29 | 2.03244% | 1.64087% | 1.01478% | 0.97528% |
| 30 | 1.84512% | 1.48909% | 0.920373% | 0.884516% |
| 31 | 1.67478% | 1.35116% | 0.834676% | 0.802131% |
| 32 | 1.51991% | 1.22585% | 0.756897% | 0.727363% |
| 33 | 1.37917% | 1.11203% | 0.686316% | 0.659517% |
| 34 | 1.25129% | 1.00867% | 0.622276% | 0.597962% |
| 35 | 1.13514% | 0.914827% | 0.564177% | 0.542121% |
| 36 | 1.02965% | 0.829642% | 0.511474% | 0.491469% |
